# Structure cristalline de type alluaudite KNa_5_Mn_3_(MoO_4_)_6_

**DOI:** 10.1107/S2056989014027030

**Published:** 2015-01-01

**Authors:** Chahira Bouzidi, Wafa Frigui, Mohamed Faouzi Zid

**Affiliations:** a Laboratoire de Matériaux et Cristallochimie, Faculté des Sciences de Tunis, Université de Tunis ElManar, 2092 Manar II Tunis, Tunisia

**Keywords:** crystal structure, alluaudite-type, hexa­kis­(molybdate)

## Abstract

The new phase potassium penta­sodium trimanganese hexa­kis(molybdate), KNa_5_Mn_3_Mo_6_O_24_, has been synthesized using solid-state methods. The structure is composed of *M*_2_O_10_ (*M* = Mn, Na) dimers and MoO_4_ tetra­hedra (point group symmetry 2) sharing corners and forming layers parallel to (100), which are linked *via* common corners of another type of *M*O_4_ tetra­hedra, forming a three-dimensional structure with two types of large channels along [001] in which two types of Na^+^ cations (one with site symmetry 2, one with -1) and K^+^ cations (site symmetry 2, half-occupation) are located. Mn^2+^ and the third type of Na^+^ cations are located at the same site *M* with occupancies of 0.75 and 0.25, respectively. A comparative structural description is provided between the structure of the title compound and those of the related phases Cu_1.35_Fe_3_(PO_4_)_3_ and NaAgFeMn_2_(PO_4_)_3_.

## Abstract 2

In the new molybdenum oxide potassium penta­sodium trimanganese hexa­kis­(molybdate), KNa_5_Mn_3_Mo_6_O_24_, the structure is composed of *M*_2_O_10_ (*M* = Mn, Na) dimers and MoO_4_ tetra­hedra sharing corners and forming layers which are linked *via* common corners of another type of MO_4_ tetra­hedra, forming a three-dimensional structure with two types of large channels in which the Na^+^ and K^+^ cations are located.

## Contexte chimique   

L’étude des matériaux à charpente ouvertes formées d’octa­èdres et de tétraèdres a connu un progrès tout à fait remarquable (Mikhailova *et al.*, 2010 ▸). En effet, on a vu naître et se développer de nombreux matériaux de ce type (Leclaire *et al.*, 2002 ▸). L’originalité de ce domaine tient en faite aux fortes relations qui existent entre son développement et ceux de ses applications notamment: conduction ionique (Sebastian *et al.*, 2003 ▸; Prabaharan *et al.*, 1997 ▸) et propriétés magnétiques (Seungdon & Seung-Tae, 2005 ▸).

Lors de l’exploration des systèmes *A*–Mn–Mo–O (*A* = ion monovalent), une nouvelle phase de formulation KNa_5_Mn_3_Mo_6_O_24_ a été synthétisée par réaction à l’état solide (température proche de 950 K). Un examen bibliographique montre que notre matériau est de type alluaudite (Kacimi *et al.*, 2005 ▸; Hatert, 2006 ▸).

## Commentaire structurelle   

**Figure 1:**
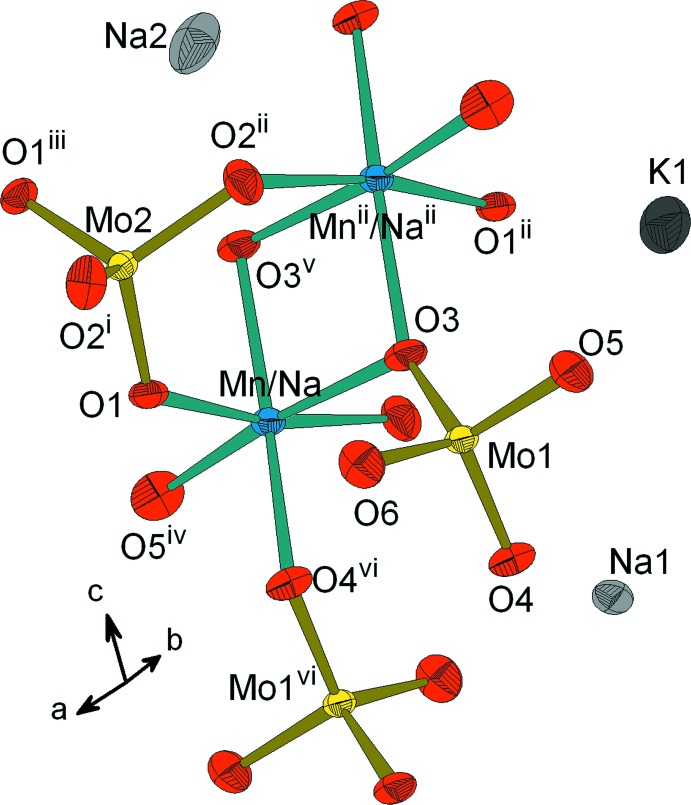
Réprésentation de l’unité structurale dans KNa_5_Mn_3_Mo_6_O_24_, mettant en évidence l’unité asymétrique et la connexion des polyèdres. Les éllipsoïdes ont été définis avec 50% de probabilité. [Codes de symétrie: (i) −*x* + 1, *y*, −*z* + 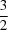; (ii) *x* − 1, *y*, *z*; (iii) −*x*, *y*, −*z* + 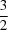; (iv) *x*, *y*, *z* − 1; (v) *x* − 1, *y*, *z* − 1; (vi) *x* − 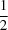, −*y* + 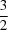, *z* − 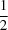.]

**Figure 2:**
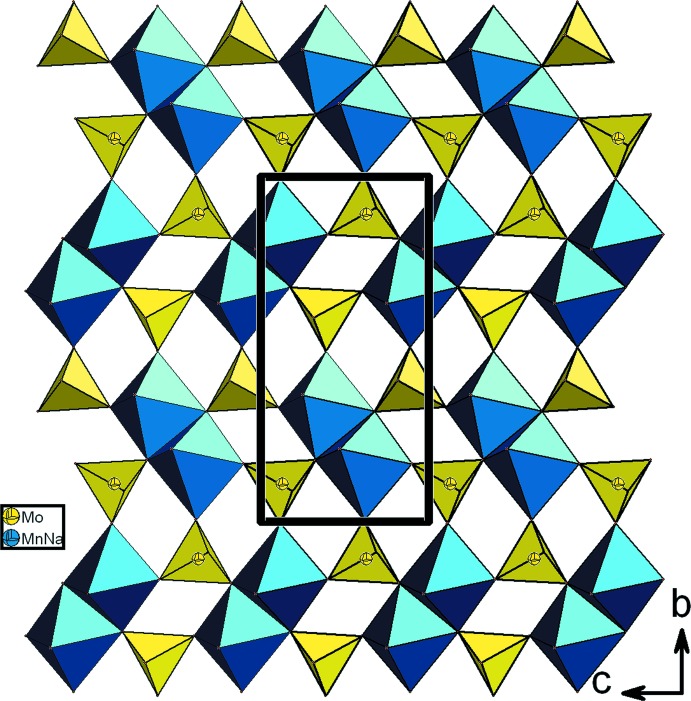
Représentation d’une couche, selon [100], dans KNa_5_Mn_3_Mo_6_O_24_.

**Figure 3:**
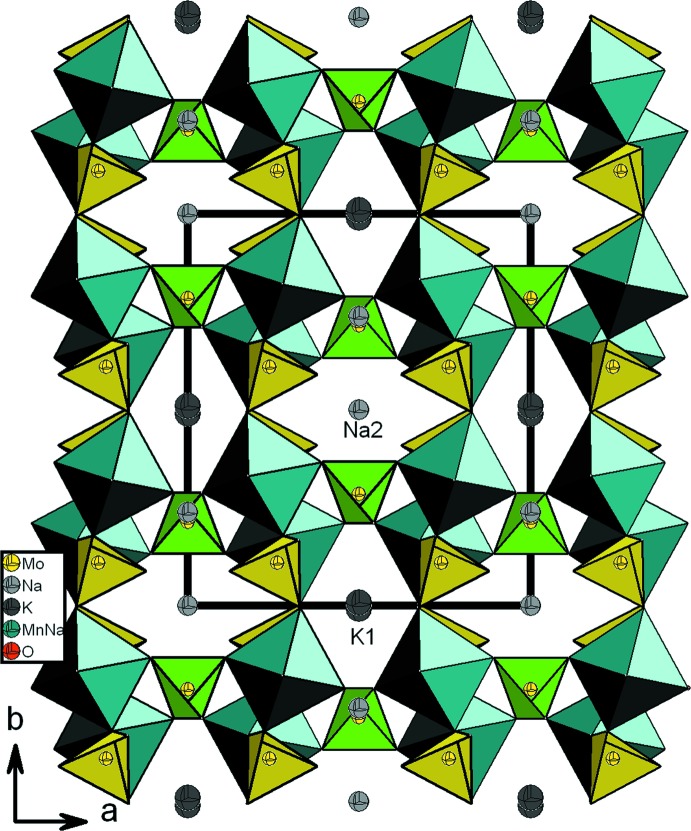
Projection de la structure de KNa_5_Mn_3_Mo_6_O_24_, selon [001].

L’unité asymétrique renferme un octa­èdre *M*O_6_ (*M* = Mn/Na) et deux tétraèdres MoO_4_ connectés par des ponts mixtes de type *M*–O–Mo. La compensation de charges dans la structure est assurée par les cations alcalins (Fig. 1 ▸). Dans la charpente anionique, les dimères *M*_2_O_10_ se lient par partage de sommets aux tétraèdres Mo1O_4_ pour conduire à des couches disposées parallèlement au plan (100) (Fig. 2 ▸). L’insertion des tétraèdres Mo2O_4_ entre les couches assurent leur jonction par formation de ponts mixtes de type Mo2–O–Mn1. Il en résulte donc une charpente anionique ouverte tridimensionnelle possédant deux types des canaux, parallèles à la direction [001], où résident les cations Na2 et K1 (Fig. 3 ▸).

**Figure 4:**
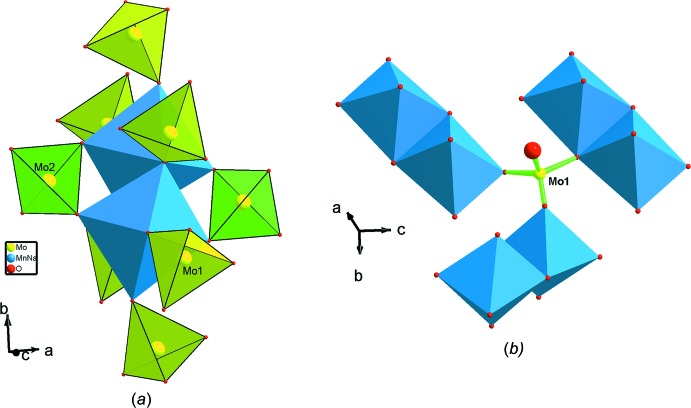
Représentation de l’environnement (*a*) d’un dimère Mn_2_O_10_ et (*b*) d’un tétraèdre Mo1O_4_.

Dans la structure chaque dimère Mn_2_O_10_ partage six de ses sommets avec respectivement six tétraèdres Mo1O_4_ différents appartenant à la même couche. Les quatre autres sommets dans chaque dimère sont mis en commun avec seulement deux tétraèdres Mo2O_4_ (Fig. 4 ▸ *a*). Il est à signaler que chaque tétraèdre Mo1O_4_ partage seulement trois de ses sommets avec trois dimères par formation de deux ponts simples et un pont triple (Fig. 4 ▸ *b*). Le quatrième sommet restant libre forme un groupement molybdyl (Mo1–O_L_) et se dirige vers le canal où logent les cations Na2. Dans la structure, chaque tétraèdre Mo2O_4_ partage ses quatre sommets avec seulement deux dimères appartenant à deux couches adjacentes (Fig. 3 ▸)

Dans les dimères *M*_2_O_10_ (*M* = Mn/Na) la distance *M⋯M* est égale à 3,429 (5) Å. Cette distance, métal–métal, courte pourrait conduire à des propriétés de super échange magnétique (Sarapulova *et al.*, 2009 ▸).

Dans le matériau, KNa_5_Mn_3_Mo_6_O_24_, les atomes de molybdène occupent totalement les sites tétraédriques (tableau 1 ▸). La moyenne des distances Mo—O égale à 1,762 (2), est conforme à celles rencontrées dans la bibliographie (Souilem *et al.*, 2014 ▸; Bugaris & zur Loye, 2012 ▸). La moyenne des distances Na—O et K—O sont égales respectivement, à 2,503 et 2,943 Å, et sont comparables à celles rencontrées dans des travaux antérieurs (Ouerfelli *et al.*, 2008 ▸; Engel *et al.*, 2009 ▸). La distance Mn1/Na3—O, égale à 2,206 Å, s’avère une moyenne des métriques Mn—O et Na—O trouvées dans d’autres structures (Chaalia *et al.*, 2012 ▸; Marzouki *et al.*, 2013 ▸).

De plus, le calcul des différentes valences de liaison (BVS), utilisant la formule empirique de Brown (Brown & Altermatt, 1985 ▸), conduit aux valeurs des charges des ions suivants: Mo1 (5,927), Mo2 (5,980), Mn1/Na3 (1,825), Na1 (1,101), Na2 (0,828) et K1 (1,005) ce qui confirme les degrés d’oxydation des différents ions attendus dans la structure.

## Enquête de base de données   

Un examen rigoureux de différentes structures trouvées dans la littérature révèle que notre matériau est de type alluaudite. Toute fois, la comparaison de notre structure avec celles de type alluaudite: Cu_1.35_Fe_3_(PO_4_)_3_ (Warner *et al.*, 1993 ▸) et NaAgFeMn_2_(PO_4_)_3_ (Daidouh *et al.*, 2002 ▸) montre qu’elles cristallisent dans le système monoclinique, présentent des paramètres de maille similaires, et ayant le même type des couches. Une différence nette dans les charpentes anioniques a été observée et en particulier dans l’arrangement atomique d’une part et le mode de connexion des couches d’autre part.

**Figure 5:**
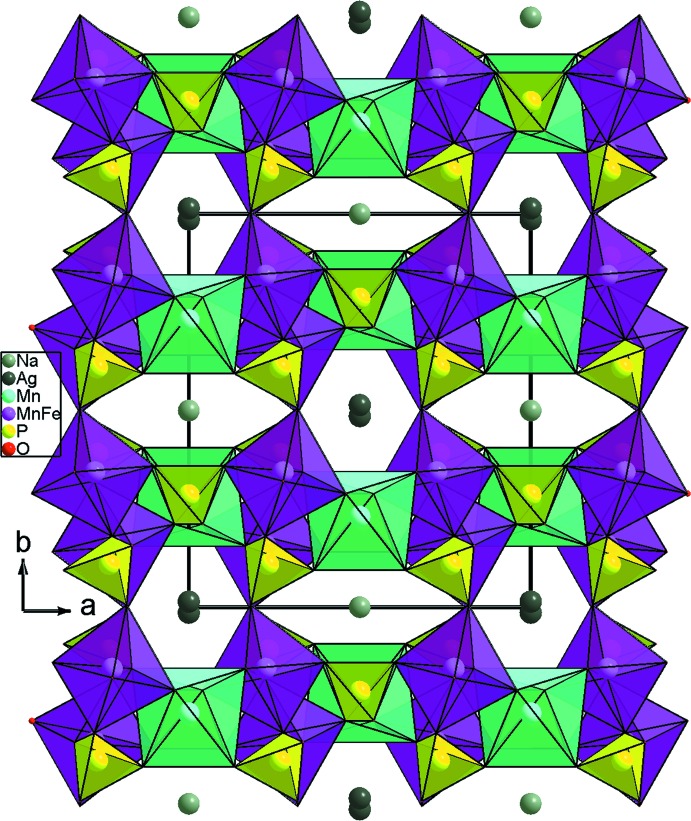
Projection de la structure de NaAgFeMn_2_(PO_4_)_3_, selon [001].

La comparaison de notre structure avec NaAgFeMn_2_(PO_4_)_3_ (Daidouh *et al.*, 2002 ▸) révèle une différence nette au niveau de l’arrangement de polyèdres. En effet, les chaînes dans NaAgFeMn_2_(PO_4_)_3_ sont construites par les octa­èdres MnO_6_ et les dimères (Fe,Mn)_2_O_10_ partageant des arêtes d’une façon alternée (MnO_6_)–[(Fe,Mn)_2_O_10_]–(MnO_6_). Par conséquent, la jonction des couches est assurée d’une part par les tétraèdres PO_4_ et d’autre part par les octa­èdres MnO_6_ pour conduire à une structure tridimensionnelle (Fig. 5 ▸).

**Figure 6:**
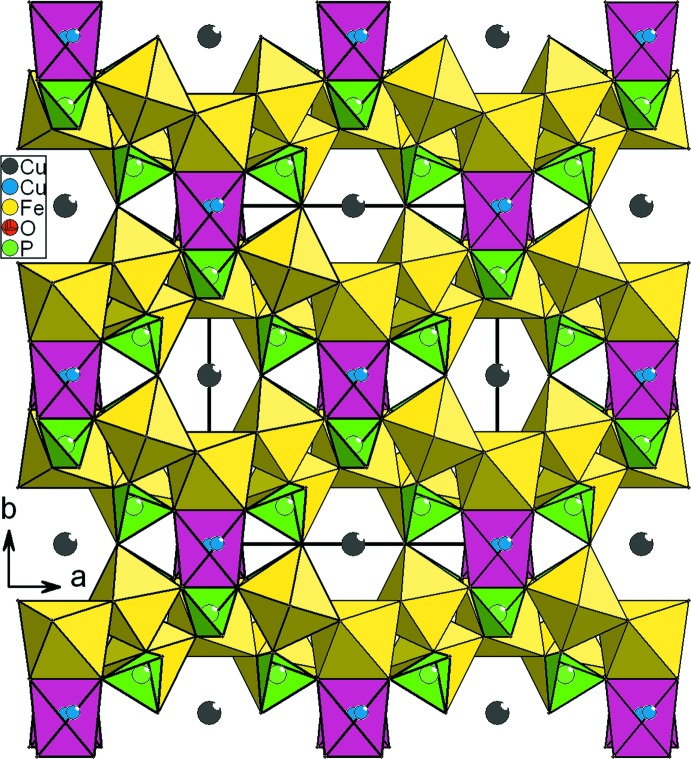
Projection de la structure de Cu_1.35_Fe_3_(PO_4_)_3_, selon [001].

Par contre, dans Cu_1.35_Fe_3_(PO_4_)_3_, les dimères Fe1_2_O_10_ sont liés par partage d’arêtes avec les octa­èdres Fe2O_6_ pour former des chaînes infinies d’octa­èdres. De plus, les couches dans Cu_1.35_Fe_3_(PO_4_)_3_ sont inter­connectés par les tétraèdres PO_4_, les polyèdres Cu(2)O_6_ et les octa­èdres Fe2O_6_ pour conduire à une structure tridimensionnelle (Fig. 6 ▸). Par contre dans le matériau obtenu KNa_5_Mn_3_Mo_6_O_24_, les couches sont connectées les unes aux autres par partage de sommets avec seulement les tétraèdres Mo2O_4_ (Fig. 3 ▸).

## Synthèse et cristallisation   

Un mélange de Na_2_CO_3_ (Prolabo, 27778) K_2_CO_3_ (Fluka, 60109), C_9_H_9_MnO_6_2H_2_O (Fluka, 63538) et (NH_4_)_2_Mo_4_O_13_ (Fluka, 69858) sont pris dans les proportions telque les rapports Na:K:Mn:Mo sont égaux à 1:1:2:3. L’ensemble est finement broyé et mis dans un creuset en porcelaine. Il est préchauffe jusqu’à 623 K afin d’éliminer les produits volatils. Le résidu a été ensuite porté à 950 K (proche de la température de fusion) et maintenu à cette dernière pendant trois semaines pour favoriser la germination et la croissance des cristaux. Un refroidissement lent (5 K/24 h) a été appliqué jusqu’à 900 K suivi d’un autre plus rapide (50 K/jour) jusqu’à la température ambiante. Des cristaux de couleur jaunâtre ont été séparés par l’eau chaude.

## Affinement   

Détails de donnés crystallines, collection de donnés et affinement sont résumés dans le tableau 2 ▸. L’affinement de tous les paramètres variables conduit à des ellipsoïdes bien définis. Les densités d’électrons maximum et minimum restants dans la Fourier-différence sont situées respectivement à 1,01 Å de Mo2 et à 1,19 Å de Mo1.

## Supplementary Material

Crystal structure: contains datablock(s) I. DOI: 10.1107/S2056989014027030/br2245sup1.cif

Structure factors: contains datablock(s) I. DOI: 10.1107/S2056989014027030/br2245Isup2.hkl

CCDC reference: 1038636

Additional supporting information:  crystallographic information; 3D view; checkCIF report

## Figures and Tables

**Table 1:** Longueurs de liaison slectionns ()

Mo1O6	1.743(3)	Na1O6^viii^	2.466(4)
Mo1O5	1.757(3)	Na1O6^ix^	2.466(4)
Mo1O4	1.762(2)	Na2O6^x^	2.531(3)
Mo1O3	1.787(3)	Na2O6	2.531(3)
Mo2O2^i^	1.759(3)	Na2O2^viii^	2.538(3)
Mo2O2^ii^	1.759(3)	Na2O2^xi^	2.538(3)
Mo2O1^iii^	1.766(2)	Na2O6^iv^	2.724(3)
Mn1O5^iv^	2.164(3)	Na2O6^i^	2.724(3)
Mn1O2^v^	2.180(3)	K1O5^i^	2.683(3)
Mn1O4^vi^	2.182(3)	K1O5^vii^	2.683(3)
Mn1O1^i^	2.191(3)	K1O5^x^	2.751(3)
Mn1O3	2.215(2)	K1O5^xii^	2.751(3)
Mn1O3^v^	2.321(3)	K1O1^xiii^	3.177(5)
Na1O1^vi^	2.367(2)	K1O1^iv^	3.177(5)
Na1O1^iii^	2.367(2)	K1O4^i^	3.184(4)
Na1O4^vii^	2.424(3)	K1O4^vii^	3.184(4)
Na1O4^i^	2.424(3)		

Codes de symtrie: (i) 
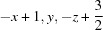
; (ii) 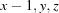; (iii) 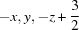; (iv) 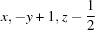; (v) 
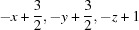
; (vi) 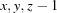; (vii) 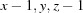; (viii) 
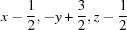
; (ix) 
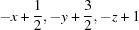
; (x) 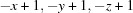; (xi) 
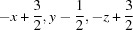
; (xii) 
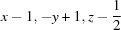
; (xiii) 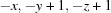.

**Table 2:** Dtails exprimentaux

Donnes crystallines	
Formule chimique	KNa_5_Mn_3_(MoO_4_)_6_
*M*_r_	1278.51
Systme cristallin, groupe d’espace	Monoclinique, *C*2/*c*
Temprature (K)	298
*a*, *b*, *c* ()	12.8943(8), 13.6295(9), 7.1809(7)
()	112.437(5)
*V* (^3^)	1166.46(16)
*Z*	2
Type de rayonnement	Mo *K*
(mm^1^)	5.05
Taille des cristaux (mm)	0.26 0.22 0.18

Collection de donnes	
Diffractomtre	EnrafNonius CAD-4
Correction d’absorption	scan (North *et al.*, 1968 ▸)
*T*_min_, *T*_max_	0.303, 0.413
Nombre de rflexions mesures, indpendantes et observes [*I* > 2(*I*)]	2955, 1266, 1155
*R*_int_	0.037
(sin /)_max_ (^1^)	0.638

Affinement	
*R*[*F* ^2^ > 2(*F* ^2^)], *wR*(*F* ^2^), *S*	0.021, 0.055, 1.13
Nombre de rflexions	1266
Nombre de paramtres	97
_max_, _min_ (e ^3^)	0.60, 0.57

Programmes informatiques: *CAD-4 EXPRESS* (Duisenberg, 1992 ▸; Macek Yordanov, 1992 ▸), *XCAD4* (Harms Wocadlo, 1995 ▸), *SHELXS97* et *SHELXL97* (Sheldrick, 2008 ▸), *DIAMOND* (Brandenburg Putz, 2001 ▸) et *WinGX* (Farrugia, 2012 ▸).

## supplementary crystallographic information

### Crystal data

**Table:**

KNa_5_Mn_3_(MoO_4_)_6_	*F*(000) = 1186
*M**_r_* = 1278.51	*D*_x_ = 3.640 Mg m^−^^3^
Monoclinic, *C*2/*c*	Mo *K*α radiation, λ = 0.71073 Å
Hall symbol: -C 2yc	Cell parameters from 25 reflections
*a* = 12.8943 (8) Å	θ = 10–15°
*b* = 13.6295 (9) Å	µ = 5.05 mm^−^^1^
*c* = 7.1809 (7) Å	*T* = 298 K
β = 112.437 (5)°	Prism, yellow
*V* = 1166.46 (16) Å^3^	0.26 × 0.22 × 0.18 mm
*Z* = 2	

### Data collection

**Table:**

Enraf–Nonius CAD-4 diffractometer	1155 reflections with *I* > 2/s(*I*)
Radiation source: fine-focus sealed tube	*R*_int_ = 0.037
Graphite monochromator	θ_max_ = 27.0°, θ_min_ = 2.3°
ω/2θ scans	*h* = −16→16
Absorption correction: ψ scan (North *et al.*, 1968)	*k* = −2→17
*T*_min_ = 0.303, *T*_max_ = 0.413	*l* = −9→9
2955 measured reflections	2 standard reflections every 120 min
1266 independent reflections	intensity decay: 1.2%

### Refinement

**Table:**

Refinement on *F*^2^	Primary atom site location: structure-invariant direct methods
Least-squares matrix: full	Secondary atom site location: difference Fourier map
*R*[*F*^2^ > 2σ(*F*^2^)] = 0.021	*w* = 1/[σ^2^(*F*_o_^2^) + (0.0194*P*)^2^ + 2.2536*P*] where *P* = (*F*_o_^2^ + 2*F*_c_^2^)/3
*wR*(*F*^2^) = 0.055	(Δ/σ)_max_ = 0.001
*S* = 1.13	Δρ_max_ = 0.60 e Å^−^^3^
1266 reflections	Δρ_min_ = −0.57 e Å^−^^3^
97 parameters	Extinction correction: *SHELXL97* (Sheldrick, 2008), Fc^*^=kFc[1+0.001xFc^2^λ^3^/sin(2θ)]^-1/4^
0 restraints	Extinction coefficient: 0.00373 (18)

### Special details

**Table:**

Geometry. All e.s.d.'s (except the e.s.d. in the dihedral angle between two l.s. planes) are estimated using the full covariance matrix. The cell e.s.d.'s are taken into account individually in the estimation of e.s.d.'s in distances, angles and torsion angles; correlations between e.s.d.'s in cell parameters are only used when they are defined by crystal symmetry. An approximate (isotropic) treatment of cell e.s.d.'s is used for estimating e.s.d.'s involving l.s. planes.
Refinement. Refinement of *F*^2^ against ALL reflections. The weighted *R*-factor *wR* and goodness of fit *S* are based on *F*^2^, conventional *R*-factors *R* are based on *F*, with *F* set to zero for negative *F*^2^. The threshold expression of *F*^2^ > σ(*F*^2^) is used only for calculating *R*-factors(gt) *etc*. and is not relevant to the choice of reflections for refinement. *R*-factors based on *F*^2^ are statistically about twice as large as those based on *F*, and *R*- factors based on ALL data will be even larger.

### Fractional atomic coordinates and isotropic or equivalent isotropic displacement parameters (Å^2^)

**Table:**

	*x*	*y*	*z*	*U*_iso_*/*U*_eq_	Occ. (<1)
Mo1	0.76468 (2)	0.61059 (2)	0.87380 (4)	0.01636 (12)	
Mo2	0.0000	0.78727 (3)	0.7500	0.01521 (13)	
Mn1	0.78663 (5)	0.65843 (5)	0.37656 (9)	0.01657 (15)	0.75
Na3	0.78663 (5)	0.65843 (5)	0.37656 (9)	0.01657 (15)	0.25
Na1	0.0000	0.76259 (17)	0.2500	0.0243 (5)	
Na2	0.5000	0.5000	0.5000	0.0539 (8)	
K1	0.0000	0.4921 (3)	0.2500	0.0512 (8)	0.50
O1	0.0426 (2)	0.7148 (2)	0.9703 (3)	0.0222 (5)	
O2	0.8937 (2)	0.8681 (2)	0.7505 (4)	0.0311 (6)	
O3	0.7807 (2)	0.6818 (2)	0.6776 (3)	0.0252 (6)	
O4	0.8271 (2)	0.6698 (2)	1.1086 (3)	0.0251 (6)	
O5	0.8292 (3)	0.4957 (2)	0.8897 (4)	0.0341 (7)	
O6	0.6219 (2)	0.5916 (2)	0.8139 (4)	0.0322 (6)	

### Atomic displacement parameters (Å^2^)

**Table:**

	*U*^11^	*U*^22^	*U*^33^	*U*^12^	*U*^13^	*U*^23^
Mo1	0.02017 (18)	0.01682 (18)	0.01267 (16)	0.00076 (11)	0.00692 (12)	−0.00067 (10)
Mo2	0.0177 (2)	0.0147 (2)	0.0122 (2)	0.000	0.00450 (15)	0.000
Mn1	0.0184 (3)	0.0184 (3)	0.0144 (3)	−0.0002 (2)	0.0079 (2)	−0.0014 (2)
Na3	0.0184 (3)	0.0184 (3)	0.0144 (3)	−0.0002 (2)	0.0079 (2)	−0.0014 (2)
Na1	0.0219 (10)	0.0333 (12)	0.0226 (10)	0.000	0.0139 (8)	0.000
Na2	0.0668 (19)	0.0312 (14)	0.0380 (14)	−0.0041 (14)	−0.0087 (13)	0.0063 (13)
K1	0.0304 (14)	0.075 (2)	0.0439 (16)	0.000	0.0090 (12)	0.000
O1	0.0223 (12)	0.0297 (14)	0.0155 (11)	0.0005 (11)	0.0082 (10)	0.0052 (11)
O2	0.0264 (13)	0.0245 (14)	0.0383 (16)	0.0039 (12)	0.0076 (12)	−0.0082 (14)
O3	0.0325 (14)	0.0305 (15)	0.0148 (11)	0.0011 (12)	0.0115 (10)	0.0013 (11)
O4	0.0346 (14)	0.0256 (14)	0.0154 (11)	−0.0060 (12)	0.0099 (10)	−0.0020 (11)
O5	0.0415 (17)	0.0255 (15)	0.0358 (15)	0.0110 (13)	0.0154 (13)	−0.0015 (13)
O6	0.0263 (14)	0.0304 (15)	0.0404 (16)	−0.0051 (12)	0.0132 (12)	−0.0045 (13)

### Geometric parameters (Å, º)

**Table:**

Mo1—O6	1.743 (3)	Na1—O4^i^	2.424 (3)
Mo1—O5	1.757 (3)	Na1—O6^viii^	2.466 (4)
Mo1—O4	1.762 (2)	Na1—O6^ix^	2.466 (4)
Mo1—O3	1.787 (3)	Na2—O6^x^	2.531 (3)
Mo2—O2^i^	1.759 (3)	Na2—O6	2.531 (3)
Mo2—O2^ii^	1.759 (3)	Na2—O2^viii^	2.538 (3)
Mo2—O1^iii^	1.766 (2)	Na2—O2^xi^	2.538 (3)
Mo2—O1	1.766 (2)	Na2—O6^iv^	2.724 (3)
Mn1—O5^iv^	2.164 (3)	Na2—O6^i^	2.724 (3)
Mn1—O2^v^	2.180 (3)	K1—O5^i^	2.683 (3)
Mn1—O4^vi^	2.182 (3)	K1—O5^vii^	2.683 (3)
Mn1—O1^i^	2.191 (3)	K1—O5^x^	2.751 (3)
Mn1—O3	2.215 (2)	K1—O5^xii^	2.751 (3)
Mn1—O3^v^	2.321 (3)	K1—O1^xiii^	3.177 (5)
Na1—O1^vi^	2.367 (2)	K1—O1^iv^	3.177 (5)
Na1—O1^iii^	2.367 (2)	K1—O4^i^	3.184 (4)
Na1—O4^vii^	2.424 (3)	K1—O4^vii^	3.184 (4)
			
O6—Mo1—O5	108.25 (14)	O2^v^—Mn1—O4^vi^	102.61 (11)
O6—Mo1—O4	110.96 (14)	O5^iv^—Mn1—O1^i^	97.38 (11)
O5—Mo1—O4	108.31 (14)	O2^v^—Mn1—O1^i^	167.33 (11)
O6—Mo1—O3	108.39 (13)	O4^vi^—Mn1—O1^i^	83.41 (9)
O5—Mo1—O3	109.94 (14)	O5^iv^—Mn1—O3	101.53 (11)
O4—Mo1—O3	110.96 (12)	O2^v^—Mn1—O3	90.23 (11)
O2^i^—Mo2—O2^ii^	102.44 (19)	O4^vi^—Mn1—O3	163.52 (11)
O2^i^—Mo2—O1^iii^	108.96 (13)	O1^i^—Mn1—O3	82.06 (10)
O2^ii^—Mo2—O1^iii^	112.10 (12)	O5^iv^—Mn1—O3^v^	171.99 (11)
O2^i^—Mo2—O1	112.10 (12)	O2^v^—Mn1—O3^v^	79.30 (10)
O2^ii^—Mo2—O1	108.96 (13)	O4^vi^—Mn1—O3^v^	89.19 (10)
O1^iii^—Mo2—O1	111.93 (18)	O1^i^—Mn1—O3^v^	89.75 (10)
O5^iv^—Mn1—O2^v^	93.99 (11)	O3—Mn1—O3^v^	83.02 (10)
O5^iv^—Mn1—O4^vi^	88.00 (11)		

Symmetry codes: (i) −*x*+1, *y*, −*z*+3/2; (ii) *x*−1, *y*, *z*; (iii) −*x*, *y*, −*z*+3/2; (iv) *x*, −*y*+1, *z*−1/2; (v) −*x*+3/2, −*y*+3/2, −*z*+1; (vi) *x*, *y*, *z*−1; (vii) *x*−1, *y*, *z*−1; (viii) *x*−1/2, −*y*+3/2, *z*−1/2; (ix) −*x*+1/2, −*y*+3/2, −*z*+1; (x) −*x*+1, −*y*+1, −*z*+1; (xi) −*x*+3/2, *y*−1/2, −*z*+3/2; (xii) *x*−1, −*y*+1, *z*−1/2; (xiii) −*x*, −*y*+1, −*z*+1.
